# Polyphasic Characterisation of Microbiota Associated with Sant’Agostino Table Olives Flavoured with *Foeniculum vulgare*

**DOI:** 10.3390/foods14213689

**Published:** 2025-10-29

**Authors:** Antonio Alfonzo, Raimondo Gaglio, Davide Alongi, Elena Franciosi, Giulio Perricone, Giuliana Garofalo, Rosario Prestianni, Vincenzo Naselli, Antonino Pirrone, Nicola Francesca, Giancarlo Moschetti, Luca Settanni

**Affiliations:** 1 Department of Agricultural, Food and Forest Sciences (SAAF), University of Palermo, Viale delle Scienze Bldg. 5 Ent. C, 90128 Palermo, Italy; antonio.alfonzo@unipa.it (A.A.); raimondo.gaglio@unipa.it (R.G.); davide.alongi@unipa.it (D.A.); giulio.perricone@unipa.it (G.P.); giuliana.garofalo01@unipa.it (G.G.); rosario.prestianni@unipa.it (R.P.); vincenzo.naselli@unipa.it (V.N.); antonino.pirrone@unipa.it (A.P.); giancarlo.moschetti@unipa.it (G.M.); luca.settanni@unipa.it (L.S.); 2 Research and Innovation Centre, Edmund Mach Foundation, Via Edmund Mach 1, 38010 San Michele all’Adige, Italy; elena.franciosi@fmach.it

**Keywords:** Illumina MiSeq, lactic acid bacteria, table olives, wild fennel, yeasts

## Abstract

Sant’Agostino green table olives, traditionally processed in Apulia and flavoured with *Foeniculum vulgare*, represent a niche product whose microbial ecology remains largely unexplored. This study aimed to characterise the microbiota of the final product (both brine and fruit) after six months of storage with wild fennel. Four production batches were analysed using a combined culture-dependent and culture-independent approach. Microbiological counts revealed variable levels of aerobic mesophilic microorganisms, yeasts, lactic acid bacteria (LAB), and staphylococci, with yeasts and LAB being predominant. Ten LAB strains were identified, including *Enterococcus faecium*, *Leuconostoc mesenteroides* subsp. *jonggajibkimchii*, *Leuconostoc mesenteroides* subsp. *cremoris*, *Leuconostoc pseudomesenteroides*, *Lactiplantibacillus plantarum*, and *Lactiplantibacillus pentosus*. Yeast isolates belonged to *Candida tropicalis*, *Torulaspora delbrueckii*, and *Saccharomyces cerevisiae*. Amplicon sequencing (MiSeq Illumina) revealed distinct bacterial profiles between fruit and brine samples, with taxa from Actinobacteria, Bacteroidetes, *Enterococcus*, *Lactobacillus*, *Leuconostoc*, Alphaproteobacteria, Enterobacteriaceae, and other Gammaproteobacteria. *Enterococcus* and *Leuconostoc* were consistently detected, while *Lactobacillus* sensu lato appeared only in one fruit and one brine sample. These findings provide new insights into the microbial diversity of Sant’Agostino olives and contribute to the understanding of their fermentation ecology and potential for quality and safety enhancement.

## 1. Introduction

From 1990 to 2023, global production of table olives has markedly increased, rising from 950,000 t to over 3000 million t [1]. In 2023, the European Union (EU) led the world in production with 825,000 t, followed by Turkey with 605,000 t and Egypt with 600,000 t. Within the EU, Spain was the top producer at 414,000 t, followed by Greece (175,000 t), Italy (62,500 t), and Portugal (25,500 t) [2]. According to the International Olive Council [3], the EU, Turkey, and Egypt were the largest consumers of table olives in 2022/23. The production and consumption volumes highlight the widespread culinary use of table olives [4]. Different olive varieties worldwide are suitable for table olive production [5]. By employing various processing methods, it is possible to produce olives with diverse colours, tastes, smells, and textures [6]. However, freshly harvested olive drupes are unpalatable due to bitterness due to the presence of oleuropein, a polyphenol that affects their edibility [7]. The two main processing methods that degrade oleuropein, rendering olives edible are the Greek (natural) method and the Spanish (Seville) method. Both methods involve microorganisms that hydrolyse oleuropein during fermentation through beta-glucosidase activity [8]. The Spanish method also includes partially debittering the olives with soda before lactic acid fermentation [9]. Various processing styles may vary according to local customs and traditions [10].

Among the traditional methods with mainly local distribution, Sant’Agostino table olives from Apulia (Cerignola, Foggia, Italy) are processed similarly to the Seville style. After a generally “spontaneous” fermentation process, the olives are packed in glass jars and flavoured with wild fennel stems and inflorescences (*Foeniculum vulgare*). The addition of spices to table olives during packing is relatively rare. Campo Real table olives, produced near Madrid, are similar and are mainly flavoured with thyme, oregano, and fennel [11]. In some recipes, Cobrançosa table olives are immersed in brine enriched with herbs like thyme, oregano, and mint after fermentation [12]. Chalkidiki green table olives, produced using the Spanish low-salt method, are occasionally preserved in olive oil infused with essential oils of various spices to control undesired microorganisms [13]. While the antimicrobial properties of wild fennel against various microorganisms are well-known [14], in oil production, *F. vulgare* extracts are sometimes used to flavour and preserve oil from oxidative processes [15].

Recent studies have demonstrated that the microbial communities involved in the fermentation of vegetables, including table olives, are influenced by ecological and technological factors and can play an active role in product preservation through the production of antimicrobial metabolites. Lactic acid bacteria and specific yeasts are recognised for their capacity to inhibit the growth of both pathogenic and spoilage microorganisms, thereby ensuring microbiological stability and promoting food safety [16,17,18]. The addition of spices and aromatic plants, such as *F. vulgare*, has been demonstrated to significantly influence the microbial composition of the fermented product. Extracts and essential oils of F. vulgare have been shown to possess significant antimicrobial activity against Gram-positive and Gram-negative bacteria, attributable to the presence of phenolic and terpenic compounds [19,20]. These interactions have also been observed in other fermented vegetables, where the use of plant ingredients with antimicrobial properties has helped to improve the microbiological stability and shelf life of the product.

The specific microorganisms present in Sant’Agostino table olives flavoured with wild fennel are not documented in the literature. Since this product undergoes “spontaneous” fermentation, the microorganisms involved can vary by season and processing environment. To improve the quality of these olives, a deeper understanding of their microbiological aspects is essential. The spontaneous fermentation of Sant’Agostino table olives traditionally takes place under consistent environmental and processing conditions, which may result in the recurrence of particular microbial groups. Nevertheless, the final microbial composition can vary depending on the native microbiota of the olives and their surrounding environment.

This study aimed to evaluate the microbial diversity of Sant’Agostino green olives flavored with wild fennel using a combined, culture-dependent and culture-independent, multi-phase approach.

## 2. Materials and Methods

### 2.1. Table Olive Processing Method and Sampling

Drupes of the Sant’Agostino olive variety were harvested in the first ten days of October 2023 while still unripe. After an initial washing in tap water to remove dust and soil residues, they were placed in a 2% (*w*/*v*) NaOH solution (Farmalabor, Canosa di Puglia, Italy) for 6–7 h. The olives were then washed five times with potable water to remove excess sodium hydroxide. Fermentation occurred in fully openable 220 L high-density polyethylene (HDPE) drums with metal tie closures (Pack Service, Liscate, Italy) and lasted one month. Each drum contained 150 kg of olives immersed in approximately 70 L of a 10% (*w*/*v*) NaCl brine solution (Sali Alimentari e Industriali SRL, Margherita di Savoia, Italy). Fermentation was carried out at ambient temperature (21 °C ± 2 °C). During the wild fennel harvest in November, the olives were transferred from the drums into 5 L glass jars with screw caps (Vetrobari SRL, Bari, Italy) in the same brine (Figure S1). Twenty g of fresh wild fennel was added to each jar. The jars were kept in a cool, dark environment for about 5 months prior to consumption (with a maximum storage duration of six months). For microbiological analysis, one jar was taken from each of four independent production batches (n = 4), all of which were processed using the same spontaneous fermentation method. The olive fruit and brine from each batch were separated and labelled P1–P4 and S1–S4, respectively. All samples were collected at the same time after six months of storage.

### 2.2. pH Measurement

The pH of the brine samples was measured at the end of the storage period using a calibrated digital pH meter (model HI98165, Hanna Instruments, Ronchi di Villafranca Padovana, Italy) [21]. Measurements were performed in triplicate at room temperature (20 ± 1 °C) immediately after opening the jars. The average pH values were recorded and used to evaluate the acidification level of the final product.

### 2.3. Microbiological Analysis

Microbiological analyses were conducted on both the olive fruit (P1, P2, P3, and P4) and the brine (S1, S2, S3, and S4). For each sample, 10 g of olive fruit and 25 mL of brine [21] were diluted 1:10 in sterile Ringer solution (Thermo Scientific™, Segrate, Italy) and homogenised for 2 min using a BagMixer^®^ 400 stomacher (Interscience, Saint Nom, France). The following microbial groups were enumerated by serial dilution and plating in Petri dishes: mesophilic aerobic microorganisms in plate count agar (PCA; Biotec, Grosseto, Italy) at 30 °C for 48 h [22]; mesophilic lactobacilli in de Man-Rogosa-Sharpe (MRS; Condalab, Torrejón de Ardoz, Spain) agar with cycloheximide (10 mg/mL) and incubated under microaerophilic conditions at 30 °C for 48 h [21]; mesophilic lactococci incubated at microaerophilic conditions on M17 agar (Condalab, Torrejón de Ardoz, Spain) supplemented with cycloheximide (10 mg/mL) at 30 °C for 48 h [23]; pseudomonads on Pseudomonas agar base (PAB; Microbiol, Cagliari, Italy) with CFC supplement (PCFC) incubated aerobically at 20 °C for 48 h [24]; staphylococci on mannitol salt agar (MSA; Microbiol, Cagliari, Italy) incubated aerobically at 32 °C for 78 h [25]; spore-forming bacteria were determined by subjecting the samples (fruit and brine) to a heat treatment in a thermostatic water bath (model 740, Asal SRL, Cernusco sul Naviglio, Italy) at 78 °C for 20 min to inactivate vegetative cells. After cooling, the samples were plated on tryptic soy agar (TSA, Condalab, Torrejón de Ardoz, Spain) and incubated at 30 °C for 24 h [26]; Enterobacteriaceae on violet red bile salt glucose agar (VRBGA; Microbiol, Cagliari, Italy) at 37 °C for 24 h [21]; coliforms in violet red bile salt agar (VRBA; Oxoid, Milan, Italy) at 37 °C for 24 h [27]; *Salmonella* spp. and *Shigella* spp. were detected using a multi-step enrichment protocol [28]. Ten g of olive pulp or 25 mL of brine were pre-enriched in 90 mL of buffered peptone water (BPW; Oxoid, Milan, Italy) and incubated at 37 °C for 18–24 h. For *Salmonella* spp., 1 mL of the pre-enrichment culture was transferred into 10 mL of Rappaport-Vassiliadis (RV) broth (Oxoid, Milan, Italy) and incubated at 42 °C for 24 h. For *Shigella* spp., a separate aliquot was inoculated into 10 mL of Gram-negative (GN) broth (Oxoid, Milan, Italy) and incubated at 37 °C for 24 h. After selective enrichment, cultures were streaked onto Hektoen Enteric Agar (HEA; Condalab, Torrejón de Ardoz, Spain) and incubated at 37 °C for 24 h; yeasts in dichloran-Rose-Bengal chloramphenicol (DRBC; Condalab, Torrejón de Ardoz, Spain) agar incubated at 25 °C for 96 h [29]. Microbial counts were expressed as log CFU/mL or g as the mean of three replicates for each sample.

### 2.4. Isolation, Phenotypic and Genotypic Characterisation of Lactic Acid Bacteria and Spore-Forming Bacteria

Bacterial colonies were collected on MRS, M17 and TSA from the highest dilution of the cell suspension of each sample from each batch. When the macroscopic morphology was found to be identical, at least three colonies were collected. These colonies were initially subjected to Gram staining [30] and the catalase test [31]. Presumptive lactic acid bacteria (LAB) were identified as Gram-positive and catalase-negative colonies isolated from MRS and M17 agar. These presumptive LAB and spore-forming isolates were purified by streaking on the same medium used for plate counting. The pure cultures (n = 80) were then transferred to the corresponding liquid medium. After incubating at 30 °C for 48 h, they were supplemented with 20% (*v*/*v*) glycerol and stored at −80 °C for further characterisation, for a maximum of 12 months. Cell morphology was observed using an Axiophot light microscope (Carl Zeiss, Oberkochen, Germany). All isolates were tested for their ability to grow at 15 °C and 45 °C. The ability of enterococci to grow at pH 9.2 and in 6.5% NaCl was also evaluated [32]. The production of CO_2_ from glucose was assessed using Durham tubes containing MRS broth, incubated at 30 °C for 48 h. Gas formation was recorded as an indicator of heterofermentative metabolism. The sporulation ability was determined using the methodology described by Manetsberger et al. [26]. Resistance to increasing NaCl concentrations (0 to 12%) was assessed in tryptone soy broth (TSB) tubes incubated at 30 °C for 48 h. The ability to grow in acidic or alkaline conditions (pH 0 to 12) was evaluated by growth tests in TSB tubes at 30 °C for 48 h. Additionally, the ability of spore-forming isolates to utilize and ferment glucose and hydrolyze starch was assessed following the method outlined by Vashist et al. [33].

Genomic DNA was extracted from all bacterial isolates using the Instagene Matrix kit (Bio-Rad, Hercules, CA, USA) after 24 h of growth in their respective optimal media. This process followed the instructions provided by the manufacturer and included cell lysis by heat treatment in the presence of a chelating resin and the subsequent removal of cell debris by centrifugation. The resulting DNA-containing supernatant could then be used directly for further investigations.

Strain typing was conducted using randomly amplified polymorphic DNA (RAPD)-PCR analysis with M13, AB106, and AB111 primers [34,35,36]. The PCR conditions and electrophoresis gel visualization followed the methods described by Alfonzo et al. [37]. RAPD profiles were analyzed using Gelcompare II software version 6.5 (Applied-Maths, Sint-Marten-Latem, Belgium). Isolates with unique patterns were identified as different strains. All bacterial strains were genetically identified by sequencing the 16S rRNA gene and comparing the sequences to public databases (GenBank and EZ-taxon) using a BLAST v2.16.0 search. PCR reactions were performed according to Allioui et al. [38] using the primer pair fD1 (5′-AGAGTTTGATCCTGGCTCAG-3′)/rD1 (5′-AAGGAGGTGATCCAGCC-3′). PCR products were purified with the QIAquick purification kit (Quiagen S.p.a., Milan, Italy) and sequenced at BMR Genomics (Padova, Italy) using the same primers as for PCR amplification, after confirming the molecular size of the amplicons (approximately 1600 bp) on agarose gels. All strains belonging to the *Lactiplantibacillus plantarum* group were subjected to the recA gene-based multiplex PCR technique described by Torriani et al. [39] with an objective to unequivocally distinguish between *L. plantarum*, *Lactiplantibacillus paraplantarum* and *Lactiplantibacillus pentosus*.

### 2.5. Isolation, Phenotypic and Molecular Characterisation of Yeasts

Yeast colonies were collected by DRBC from the highest dilution of the cell suspension of each sample in each lot. A minimum of three colonies were collected if the macroscopic morphology appeared similar. These colonies were cultured for 3 d at 25 °C in test tubes containing yeast peptone dextrose (YPD) broth (Condalab, Torrejón de Ardo, Spain) and purified by streaking on the same agar medium. The purity of the isolates was verified microscopically. Pure colonies (n = 54) were then stored at −80 °C in 20% (*v*/*v*) glycerol YPD broth for further characterization, for a maximum of 12 months. The axenic yeast isolates were initially differentiated by observing the following characteristics after growth on Wallerstein Laboratory (WL) nutrient agar (25 °C, 5 days): colony colour, shape, rim, opacity, surface, and consistency [40,41,42]. Genomic DNA was extracted from all isolates using the same method as for bacterial isolates. The extracted DNA was amplified for 5.8S rRNA using ITS1 and ITS4 primers [43]. Preliminary species identification was achieved through Restriction Fragment Length Polymorphism (RFLP) analysis by generating restriction profiles through DNA digestion with the enzymes C*fo*I, H*ae*III, and H*inf*I (Thermo Fisher Scientific, Monza, Italy) following the methodology of Sinacori et al. [44]. Band visualization was performed as described by Alfonzo et al. [45]. Isolates with identical RFLP profiles were further distinguished into strains using DNA (RAPD)-PCR analysis with M13 primer [46], following the amplification conditions, band visualization, and RAPD profile analysis reported by Alfonzo et al. [45]. To confirm the identification at species level determined by RFLP analysis, each yeast strain was subjected to sequencing of the D1/D2 region of the 26S rRNA gene with primers NL1 and NL4, following the methodology reported by Kurtzman and Robnett [47]. Sequence verification was manually performed using Chromas 2.6.6 software (Technelysium Pty Ltd., Brisbane, Australia) after DNA sequencing at BMR Genomics (Padova, Italy). The sequence of each yeast strain was entered into the BLAST search engine to compare the percentage similarity with sequences in the GenBank database (https://blast.ncbi.nlm.nih.gov/Blast.cgi, accessed on 13 November 2024).

### 2.6. Extraction of the DNA and Preparation of the MiSeq Library

Total DNA was extracted from each table olive sample by separating the fruit from the brine using the NucleoSpin Food kit (Macherey-Nagel GmbH & Co. KG, Düren, Germany) following the manufacturer’s protocol. For each sample (both fruit and brine), a 464 bp fragment of the V3–V4 region [48,49] of the 16S rRNA gene (corresponding to *Escherichia coli* positions 341 to 805) was amplified from the extracted DNA. PCR amplification of each sample was performed in 25 μL of reaction volume, with 12.5 μL of 2× KAPA Hifi HotStart Ready Mix (Kapa Biosystems Ltd., London, UK), 1 μM of each primer, 2 μL of DNA (10 ng/μL), and 9.5 μL of ddH20. All PCR reactions were carried out using a Verity™ 96-well Thermal Cycler, according to the following protocol: 95 °C for 5 min and 25 cycles of 95 °C for 30 s, 55 °C for 30 s, 72 °C for 40 s, with a final elongation step of 72 °C for 5 min. PCR products were checked by gel electrophoresis and cleaned using an Agencourt AMPure XP system (Beckman Coulter, Brea, CA, USA), following the manufacturer’s instructions. After seven PCR cycles (16S metataxonomic Sequencing Library Preparation, Illumina), Illumina adaptors were attached (Illumina Nextera XT Index Primer). Libraries were purified using Agencourt AMPure XP (Beckman Coulter, Brea, CA, USA) and then sequenced on an Illumina^®^ MiSeq (Run Chemistry: 2 × 300 PE) platform (MiSeq Control Software 2.0.5 and Real-Time Analysis software 1.16.18, Illumina, San Diego, CA, USA). Amplicon library preparation, quality and quantification of pooled libraries and high-throughput sequencing by Illumina technology were performed at the Sequencing Platform, Fondazione Edmund Mach (FEM, San Michele all’ Adige, TN, Italy).

### 2.7. Illumina Data Analysis and Sequences Identification

The raw paired-end FASTQ files were demultiplexed using idemp (available at https://github.com/yhwu/idemp/blob/master/idemp.cpp, accessed on 13 November 2024). Subsequently, they were imported into Quantitative Insights Into Microbial Ecology (Qiime2, version 2018.2). The sequences underwent quality filtering, trimming, de-noising, and merging using DADA2 [50]. Briefly: forward and reverse reads with a total number of expected errors of >2 were discarded. Next, the DADA2 workflow was used to trim quality-controlled reads to specific lengths, to identify exact sequences with single-nucleotide resolution, and to filter de novo chimeras with consensus method.

Taxonomic and compositional analyses for bacteria were conducted using the feature-classifier plugin (available at https://github.com/qiime2/q2-feature-classifier, accessed on 13 November 2024) [51]. A pre-trained Naive Bayes classifier, based on the Greengenes 13_8 99% Operational Taxonomic Units (OTUs) database (previously trimmed to the V3–V4 region of 16S rDNA and bound by the 341F/805R primer pair), was used to generate taxonomy tables from paired-end sequence reads. The MiSeq Illumina sequencing data have been deposited in the NCBI Sequence Read Archive (SRA) and are accessible under Accession Number PRJNA1185664 [52].

### 2.8. Statistical Analysis

The microbial count data were subjected to analysis of variance (ANOVA) and compared pairwise using Tukey’s post hoc test. Statistical significance was set at *p* ≤ 0.001. The software used for statistical data processing was XLStat software ver. 2019.2.2 (Addinsoft, New York, NY, USA).

## 3. Results

### 3.1. pH Values of Brine Samples

The pH values measured in the brine samples at the conclusion of the storage period were 4.38 ± 0.04 (S1), 4.34 ± 0.06 (S2), 4.30 ± 0.03 (S3), and 4.35 ± 0.02 (S4), respectively. These values indicate the extent of acidification achieved during the fermentation process across the four production batches.

### 3.2. Microbial Counts

The results of the microbial count (Table 1) revealed the presence of key microbial groups characteristic of table olives.

Variations were noted among the different samples regarding total mesophilic microorganism count, yeasts, rod LAB, and staphylococci. Conversely, no significant differences were observed for cocci-shaped LAB. Pseudomonads, Enterobacteriaceae and coliforms were not detected in any of the samples by direct enumeration, while the absence of *Salmonella* spp. and *Shigella* spp. was confirmed through enrichment-based methods. Higher levels of total mesophilic microorganisms were found in the brines (S2, S3, and S4), ranging from 6.8 to 6.9 log CFU/mL. Similarly, yeast counts were higher in the brine samples (5.6–5.9 log CFU/mL) compared to the fruit samples (4.5–4.9 log CFU/g). Sample P4 had the lactobacilli count (7.3 log CFU/g), while S1 had the lowest (6.0 log CFU/mL). There was low variability between brine and fruit samples for LAB cocci populations, with counts ranging from 5.8 log CFU/mL (S1) to 6.7 log CFU/g (P4). Spore-forming bacteria were only found in brine samples (S1, S2, S3, and S4), with microbial concentrations of approximately 2.5 log cycles. Staphylococcaceae levels were higher in brine samples, ranging from 5.2 to 5.9 log CFU/mL, whereas fruit samples showed values between 2.7 and 4.4 log CFU/g.

### 3.3. Phenotypic Grouping and Genetic Identification

#### 3.3.1. Lactic Acid Bacteria

Colonies with different macroscopic characteristics in Petri dishes used to enumerate rod-shaped (MRS) and coccus-shaped (M17) LAB in both brine and olive fruit were subjected to purification and phenotypic grouping. A total of 62 isolates, characterised by Gram positivity and catalase negativity, were identified as presumptive LAB. Microscopic observations revealed that the most common cell morphology was coccus, observed in 58 isolates, with short chains present in 22 isolates. Only four isolates exhibited a rod-shaped morphology. Preliminary physiological and biochemical characterisation (Table 2) enabled differentiation of the coccus-shaped isolates into four distinct groups, while the rod-shape morphology was represented by a single phenotypic group.

**Figure 1:**
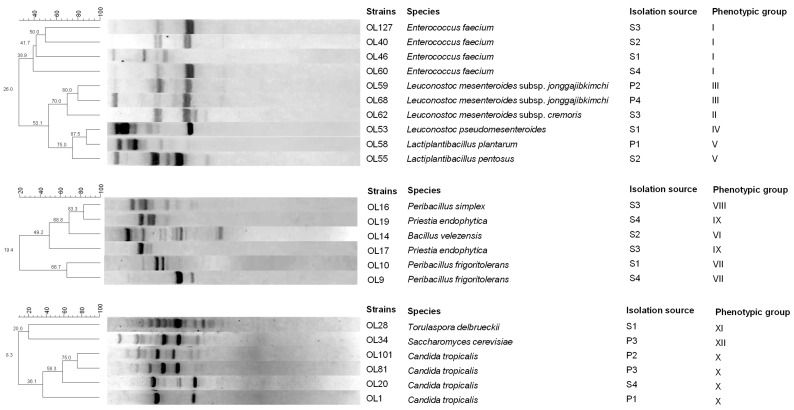
Dendrogram obtained for LAB, spore-forming bacteria and yeast strains isolated from the brine and fruit of Sant’Agostino table olives using RAPD-PCR. The top line indicates the percentage of similarity. Acronyms: S1–S4, brine from 4 production batches; P1–P4, fruit from 4 different production batches.

The presumptive LAB were subjected to RAPD-PCR analysis to differentiate them at the strain level. A comparison of the polymorphic profiles allowed the differentiation of 10 strains (Figure 1), which were subsequently identified to the species level by sequencing the 16S rRNA gene.

Species-specific and multiplex PCRs were also used to analyse the species within the *L. plantarum* group. In total, six species were identified (Table 3): *Enterococcus faecium* (n = 4); *Leuconostoc mesenteroides* subsp. *jonggajibkimchii* (n = 2); *Leuconostoc mesenteroides* subsp. *cremoris* (n = 1); *Leuconostoc pseudomesenteroides* (n = 1); *L. plantarum* (n = 1); and *L. pentosus* (n = 1).

#### 3.3.2. Spore-Forming Bacteria

Isolates of spore-forming bacteria found only in the brine samples were positive for Gram stain and catalase tests and exclusively exhibited rod cell morphology. Based on their physiological and biochemical characteristics (Table 2), they were divided into four groups. RAPD-PCR analysis differentiated the 18 isolates into six strains (Figure 1). Each strain was then identified at the species level by sequencing the 16S rRNA region: *Peribacillus simplex* (n = 1); *Priestia endophytica* (n = 2); *Bacillus velezensis* (n = 1); and *Peribacillus frigoritolerans* (n = 1).

#### 3.3.3. Yeasts

Fifty-four yeast isolates were classified into three phenotypic groups (Table 4) based on the macroscopic characteristics of the colonies.

Group VI had the largest number of isolates (n = 50), followed by group VIII (n = 3), and group VII (n = 1). Common characteristics included a rounded colony shape and a butyrous consistency. Colony colour facilitated phenotypic discrimination of the groups, with additional differences observed in edge, opacity, and surface characteristics. RFLP analysis presumptively identified the yeast isolates as belonging to three species: *Candida tropicalis*, *Torulaspora delbrueckii*, and *Saccharomyces cerevisiae* (Table 5).

RAPD-PCR analysis further differentiated isolates with the same RFLP profile at the strain level. The 54 yeast isolates comprised six strains: four of *C. tropicalis*, and one each of *T. delbrueckii* and *S. cerevisiae*. Sequencing of the D1/D2 region of the 26S rRNA gene and comparison with the sequences in Genbank confirmed the species identifications obtained by RFLP analysis.

### 3.4. Cultivable LAB, Spore-Forming Bacteria and Yeast Species Distribution

The species distributions of microorganisms isolated from the brine and olive fruit samples are reported in Table 6.

The highest number of microbial species was observed in the brine samples S1 and S3 (six species), while the lowest number of species was recorded in fruit sample P1 (three species). Both brine and fruit samples revealed the presence of several LAB species, including *E. faecium* (P2–P4, S1–S4), *L. plantarum* (P1, S3), *Ln. mesenteroides* subsp. *cremoris* (P1, P3 and S1–S3), and *Ln. mesenteroides* subsp. *jonggajibkimchii* (P2–P4 and S4). LAB species found only in brine samples were *L. pentosus* (S2) and *Ln. pseudomesenteroides* (S1). Spore-forming bacteria were detected only in the brine samples. Specifically, *P. frigoritolerans* and *Pr. endophytica* were found in S1 and S4, and S3 and S4, respectively. *Bacillus velezensis* was present in S2 sample, and *P. simplex* in S3.

*Candida tropicalis* was confirmed in all brine and fruit samples. In contrast, *S. cerevisiae* was identified only in three fruit samples (P2, P3, and P4), and *T. delbrueckii* was isolated solely from brine sample S1.

### 3.5. Characterisation of the Illumina Data and Taxonomic Analysis of the Bacterial Community

**Figure 2:**
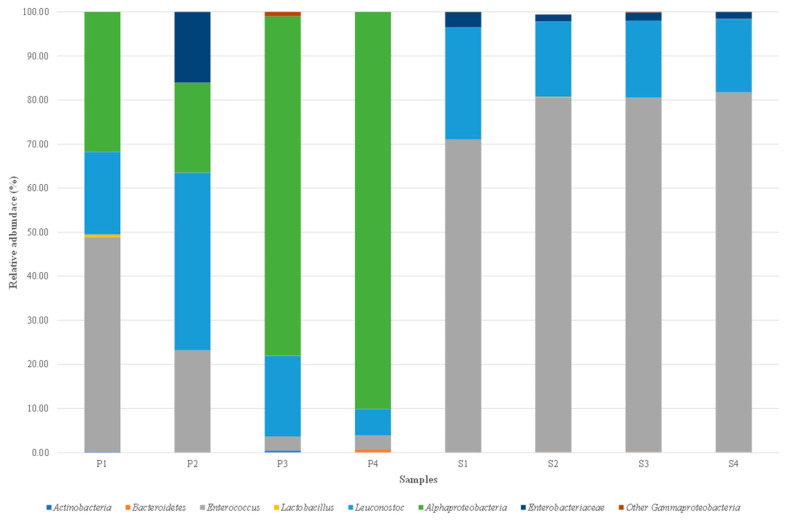
Relative abundance (%) of bacterial genera identified by MiSeq Illumina on Sant’Agostino table olives aromatised with *Foeniculum vulgare*. Only genera with abundance > 0.1% in at least one sample were included. Acronyms: S1–S4, brine from 4 production batches; P1–P4, fruit from 4 different production batches.

Illumina technology enabled identification of non-culturable (dormant and/or viable) bacterial populations in the brine and olive fruit samples. The total DNA extracted from the eight samples was successfully amplified targeting the bacterial V3–V4 region of the 16S rRNA gene. The distribution of bacterial taxa in the samples is shown in Figure 2. Eight bacterial taxa were characterised in fruit and brine samples: Actinobacteria, Bacteroidetes, *Enterococcus*, *Lactobacillus*, *Leuconostoc*, Alphaproteobacteria, Enterobacteriaceae, and other Gammaproteobacteria.

The LAB group genera *Enterococcus* and *Leuconostoc* were present in all brine and olive fruit samples, with relative abundances varying according to sample type. For *Enterococcus*, percentages in the fruit ranged from 3.19% (P4) to 48.69% (P1), and higher percentages in the brine ranged from 71.01% (S1) to 81.67% (S4). For *Leuconostoc*, percentages ranged from 6.05 to 40.33% in the fruit and 16.72 to 25.51% in the brine. The genus Lactobacillus was found in one fruit sample (P1) and one brine sample (S2), but exhibited lower relative abundance than *Enterococcus* and *Leuconostoc*, ranging from 0.11% in S2 to 0.54% in P1. The Alphaproteobacteria class was highly representative in all the olive fruit samples, with relative abundances from 20.49% (P2) to 90.12% (P4), but was low in the brine sample S2 (0.11%). Enterobacteriaceae family was present in all brine samples, with percentages from 1.55% (S4) to 3.41 (S1), and was only found in fruit sample P2 (16.03%). Other bacterial taxa were present in low relative abundances and were occasionally detected in various fruit and brine samples. Specifically, Gammaproteobacteria had the highest percentages in P3 (0.98%), Bacteroidetes in P4 (0.65%), and Actinobacteria in P3 (0.42%).

## 4. Discussion

This research assessed the microbial diversity of bacterial populations in Sant’Agostino green table olives flavoured with *F. vulgare* using both culture-dependent and culture-independent methods. While DNA-based independent approaches have recently been employed to study the microbial communities in table olives [53], combining these with culture-dependent methods enabled the assessment of the viability of the identified taxa. The cultivable microbiota of table olives mainly consists of lactic acid bacteria and yeast populations [54]. However, the microbial groups characterizing spontaneously fermented table olives are diverse and influenced by several factors. These include olive variety [55], processing location [56], pH, salinity, the amount of fermentable sugars, fermentation temperature, water activity, and oxygen availability [57], and the processing style [58].

The Sant’Agostino cultivar is well-suited to table olive processing due to its firm fruit, low oil content, and sufficient fermentable sugars [59]. Numerous studies have examined the microbiota of various table olive varieties such as Bella di Cerignola, Nocellara Etnea, Tonda di Cagliari, Cipriota, Kalamata, Picual, Gordal, Hojiblanca, and Manzanilla [7,55,58,60,61,62]. However, information on the microbiota cultivar Sant’Agostino is limited. To better understand the microorganisms associated with Sant’Agostino table olives, a study was conducted to examine the ecology of microbial groups characterizing these olives, processed following a traditional Apulian protocol. Microbiological counts on the final product identified the microbial groups present in Sant’Agostino table olives flavoured with F. vulgare. LAB and yeasts were the dominant groups, consistent with other table olive varieties processed differently [63,64,65]. Notably, all four production batches’ brines and pulp samples were free of pseudomonads, typically associated with microbial spoilage. *Pseudomonas* spp. exhibit proteolytic activity, causing a decrease in brine acidity and drupe swelling [66]. Although commonly associated with Greek and Seville table olives [66] and other methods like Castelvetrano [45], their absence may be due to the addition of *F. vulgare* during processing. Extracts or essential oils from wild fennel seeds and plant parts released into the brine during immersion have antimicrobial activity against various pathogenic and spoilage bacteria, including pseudomonads [67,68,69]. Although fresh wild fennel was added after fermentation was complete, its antimicrobial properties may have contributed to shaping the final microbial composition. The consistent absence of pseudomonads and other spoilage microorganisms across all samples could be partially attributed to the addition of wild fennel. However, as wild fennel was introduced post-fermentation and no control batches without fennel were included in the study, its specific impact cannot be conclusively determined. Further investigations comparing batches with and without fennel are required to clarify its role in modulating the microbiota of the final product.

The absence of Enterobacteriaceae and coliforms in the final product is attributable to the regular acidification process of the brine, which creates an unfavourable environment for their proliferation; in fact, the pH values of the brine in the different samples varied between 4.30 (S3) and 4.38 (S1). This results from the combination of low pH values and adequate salt content. Enterobacteriaceae and coliforms are commonly present in the early stages of the process, coinciding with high pH levels. However, during fermentation, as the brine’s pH decreases, their concentrations drop below detectable levels [70]. Elevated levels of Enterobacteriaceae and coliforms early in fermentation can irreversibly affect the quality of the final product, leading to off odours [71]. The absence of *Shigella* spp. and *Salmonella* spp. is also indicative of a regular fermentation process. Additionally, the presence of phenolic and oleosidic compounds in the brine, which have antibacterial effects, combined with a low pH and adequate salt content, creates conditions hostile to these foodborne pathogens [72]. Staphylococcaceae were the only spoilage/pathogenic bacteria present in the four production batches due to their tolerance to acidic pH and high salinity [73]. The microbial levels for Staphylococcaceae are comparable to those observed in other studies on green olives, such as Aloreña de Málaga [74], Manzanilla and Gordal [56]. However, the absence of *Staphylococcus aureus* colonies indicates that processing was carried out with appropriate hygienic conditions [75].

Investigations into the microbial groups revealed the presence of *L. plantarum* and *L. pentosus* among LAB, frequently isolated from various table olive varieties [64]. Strains of both species have been characterized and successfully employed as starter cultures to enhance the fermentation process in Nocellara del Belice [76], Cobrançosa [77], Campiñesa [78], Nocellara Etnea [79] and Manzanilla [80] table olives. However, *E. faecium* is the most prevalent LAB species in the four production batches of Sant’Agostino table olive. A study of LAB from Cypriot green table olives [81] found that the enterococci group was predominant, with all identified strains belonging to *E. faecium*. These strains demonstrate potential for application in the food industry due to their good resistance to low pH values and bile salts. El Issaoui et al. [82] identified several *E. faecium* strains from Moroccan table olives that grew at low temperatures, tolerated acidic pH and salinity, and exhibited antimicrobial activity against common pathogens. Other identified LAB species belonged to the genus *Leuconostoc*. Portilha-Cunha et al. [10] describe how *Leuconostoc*, along with *Lactobacillus*, *Pediococcus*, *Enterococcus*, and *Streptococcus*, is commonly found in table olives. Using *Ln. mesenteroides* as a starter culture, in combination with different yeast species, has shortened the brine acidification period and improved the sensory quality of the final product [83]. Maoloni et al. [84] used *Ln. pseudomesenteroides* (strain PB288) in a multi-strain starter culture to optimize the fermentation of Ascolana Tenera table olives flavoured with sea fennel (*Crithmum maritimum* L.). *Leuconostoc mesenteroides* subsp. *jonggajibkimchii*, a dominant LAB species found in kimchi [85], has not previously been reported in fermented table olives. To our knowledge, this is the first documented presence of this subspecies in such products. Additional research and repeated isolations will be necessary to clarify its ecological role and persistence in this food matrix. However, it has also been reported as the dominant fermenting agent in the Turkish vegetable drink Şalgam [86]. *Leuconostoc mesenteroides* subsp. *cremoris* is typically associated with the fermentation of dairy products, for which it is used as a starter culture [87]. Its presence in table olives has already been documented by Kumral et al. [88], and the species has been isolated from olive fruit. The present study adds to the existing evidence of its presence in fermented vegetables by detecting it in wild fennel-flavoured Sant’Agostino olives, thereby supporting the hypothesis of its adaptability to different fermentation environments.

In the group of spore-forming bacteria found exclusively in the brine samples of all four batches, none of the identified species have been previously associated with table olives. *Peribacillus frigoritolerans* was isolated from leaf samples in Andalusian olive groves [26]. *Priestia endophytica* is a spore-forming endophytic bacterium known to colonise various agricultural plants [89,90,91]. Its presence in fermented table olives has not previously been documented, but it may be associated with the use of wild fennel or water sources. Hussein et al. [92] identified *P. endophytica* strains with plant-growth-promoting (PGP) properties from barley seeds, which, when inoculated into maize plantations, improved productivity [93]. Similarly, *P. simplex*, a spore-forming bacterium that was previously isolated from olive tree environments in Spain [26], is known for its PGP properties in various crops [94]. This is the first report of its presence in fermented table olives, suggesting possible transfer from plant material, such as wild fennel, or from water used during processing.

Additionally, *B. velezensis* is a well-researched biocontrol agent effective against several fungal diseases, including verticilliosis, fusariosis, and root rot in olive trees [95,96,97]. The presence of these spore-forming bacterial species in the brine of Sant’Agostino table olives may be attributed to the washing water used to remove soda residues or the water used to produce the brine. Alternatively, their endophytic nature suggests their presence in the stems, leaves, or seeds of wild fennel used as a flavouring agent [91]. However, none of these spore-forming bacteria has been identified as a spoilage agent of fermented plant products. *Priestia endophytica* is rarely associated with infections in immunocompromised patients or those with orthopedic complications [98].

The predominant yeast species was *C. tropicalis*, frequently found in both Seville table olives [99] and Greek-style table olives [100] that undergo “spontaneous” fermentation. While the presence of yeasts in table olives was once seen negatively [101], this perspective has evolved. Selected yeast strains are currently employed, often in combination with LAB, to improve the fermentation process [102,103]. Some *C. tropicalis* strains from fermenting table olives have shown significant probiotic potential, suggesting their use in producing probiotic table olives [104,105]. *Saccharomyces cerevisiae*, known for its fermentative capacity, is used as a starter in bakery and alcoholic beverage production [106]. Its presence in table olives has been detected in several spontaneously fermented products [107,108]. Tufariello et al. [109] used a *S. cerevisiae* strain as a starter in Manzanilla, Picual, and Kalamàta table olives, reducing fermentation time and standardizing sensory and nutritional qualities. Other studies highlight its probiotic potential and application in new probiotic food products [104,105]. Combining *S. cerevisiae* with olive leaf extracts in Spanish-style table olives has enhanced fermentation performance and increased hydroxytyrosol levels, an antioxidant with anti-inflammatory, anticarcinogenic, and neuroprotective properties [110]. Unlike *C. tropicalis* and *S. cerevisiae*, *T. delbrueckii* has been occasionally isolated in Sant’Agostino table olives flavored with wild fennel. Psani and Kotzekidou [111] identified *T. delbrueckii* in Greek-style black table olives, which could degrade oleuropein and showed antimicrobial activity against *Listeria monocytogenes*, *Bacillus cereus* and *Salmonella* Typhimurium. Recently, Mujdeci and Ozbas [112] isolated *T. delbrueckii* from salted Gemlik black table olives, along with *Pichia membranifaciens*, *Candida sorbosivorans*, *Citeromyces nyonsensis*, *Candida etchelsii*, *Wickerhamomyces subpelliculosus*, *Candida apicola*, *Wickerhamomyces anomalus*, and *Candida versatilis*, which were dominant in this production process. However, no studies to date have investigated *T. delbrueckii* as a starter or probiotic strain in table olives.

Next-generation sequencing (NGS) using Illumina technology was employed to survey the microbial diversity in the production of Sant’Agostino green olives flavoured with wild fennel. NGS tools allowed for the detection of viable non-culturable microorganisms and dead cells, provided the DNA was accessible [113]. Specifically, the bacterial composition of samples from the four production batches was analyzed in both brine and fruit by amplifying the V3-V4 rRNA gene region. The *Enteroccoccus* genus present in all fruit and brine samples, similar to other table olive productions where its abundance varied with fermentation times. Notably, *Enterococcus* was found in Sevillian-style processed Manzanilla [7] and Hojiblanca [114] olives. For the genus *Leuconostoc*, despite lower relative abundance, its presence was noted in Greek or natural-style olives of the Cypriot, Kalamata, and Picual cultivars that underwent “spontaneous” fermentation [55]. The difference observed between the culture-dependent and culture-independent results, particularly regarding the dominance of *Enterococcus faecium* in culture-based analyses versus the co-dominance of *Enterococcus* and *Leuconostoc* in the MiSeq Illumina data, reflects the methodological distinctions between the two approaches. Similar discrepancies have been reported in studies on table olives, where culture-based methods tend to favour the recovery of viable and fast-growing microorganisms, while metataxonomic techniques provide a broader overview of the microbial community, including viable but non-culturable (VBNC) organisms and DNA from dead cells [73]. These differences highlight the complementarity of the two approaches, with culture-dependent methods offering insights into the technological potential of isolates, and sequencing-based methods enhancing our understanding of microbial diversity and structure. Contrary to several publications, the genus Lactobacillus showed lower relative abundance in various Greek- and Seville-style olive varieties [7,55,115,116]. Alphaproteobacteria were present in all fruit samples with an average relative abundance of 55%, compared to lower percentages found by Michailidou et al. [117] and Mougiou et al. [53] in Greek-style table olives. However, the NGS approach did not detect spore-forming bacteria in the brine samples from different production batches. It is plausible that these bacteria were present in low abundance, and the DNA amount was insufficient for NGS detection, favouring the most abundant taxa [118].

## 5. Conclusions

This study aimed to characterise both the cultivable and non-cultivable microbiota of Sant’Agostino table olives, subjected to a traditional fermentation process, with wild fennel added as a flavouring agent at the final stage. The final product, resulting from spontaneous fermentation, exhibited considerable microbial diversity. The predominant LAB species was *E. faecium*, while *C. tropicalis* was the most frequently isolated yeast. The presence of *Enterococcus* in all samples was corroborated by culture-independent analyses. The absence of viable pathogenic microorganisms indicates that, despite the spontaneous nature of the fermentation and the traditional production protocol, the four tested batches are unlikely to pose a microbiological risk to consumers. Although the number of production batches analysed was limited, further studies are warranted to comprehensively assess the microbiota associated with this specific table olive production process. The selection of starter and/or probiotic cultures of lactic acid bacteria and yeasts could further enhance microbiological control and standardise the fermentation process of Sant’Agostino olives flavoured with wild fennel.

## Figures and Tables

**Table 1:** Microbial counts of different groups of microorganisms determined on 4 different batches of Sant’Agostino olives.

Samples	Microbial Concentration (log CFU/g or CFU/mL)									
	**PCA**	**DRBC**	**MRS**	**M17**	**TSA**	**PAB**	**MSA**	**VRGBA**	**VRBA**	**EHA**
P1	4.9 ± 0.5 b	4.9 ± 0.5 abc	6.5 ± 0.1 ab	6.6 ± 0.5 a	<2.0 b	<2.0 a	3.9 ± 0.2 c	<1.0 a	<1.0 a	<1.0 a
P2	4.7 ± 0.4 b	4.5 ± 0.4 c	6.7 ± 0.2 ab	6.6 ± 0.1 a	<2.0 b	<2.0 a	4.4 ± 0.3 bc	<1.0 a	<1.0 a	<1.0 a
P3	4.8 ± 0.2 b	4.6 ± 0.4 bc	6.6 ± 0.4 ab	6.6 ± 0.4 a	<2.0 b	<2.0 a	3.0 ± 0.4 d	<1.0 a	<1.0 a	<1.0 a
P4	4.9 ± 0.3 b	4.7 ± 0.2 bc	7.3 ± 0.3 a	6.7 ± 0.1 a	<2.0 b	<2.0 a	2.7 ± 0.5 d	<1.0 a	<1.0 a	<1.0 a
S1	5.7 ± 0.5 b	5.6 ± 0.4 abc	6.0 ± 0.3 b	5.8 ± 0.4 a	2.5 ± 0.3 a	<2.0 a	5.2 ± 0.5 ab	<1.0 a	<1.0 a	<1.0 a
S2	6.8 ± 0.3 a	5.8 ± 0.5 a	6.4 ± 0.5 ab	6.3 ± 0.5 a	2.5 ± 0.3 a	<2.0 a	5.6 ± 0.2 a	<1.0 a	<1.0 a	<1.0 a
S3	6.8 ± 0.5 a	5.7 ± 0.3 ab	6.3 ± 0.1 b	6.3 ± 0.3 a	2.5 ± 0.4 a	<2.0 a	5.3 ± 0.4 a	<1.0 a	<1.0 a	<1.0 a
S4	6.9 ± 0.2 a	5.9 ± 0.4 a	6.3 ± 0.5 b	6.3 ± 0.4 a	2.6 ± 0.2 a	<2.0 a	5.9 ± 0.3 a	<1.0 a	<1.0 a	<1.0 a
Statistical significance	***	**	*	n.s.	***	n.s.	***	n.s.	n.s.	n.s.

Note: Results indicate mean values ± SD from three determination. Abbreviations: PCA, plate count agar for mesophilic aerobic microorganisms; DRBC, dichloran-Rose-Bengal chloramphenicol agar for yeast; MRS, de Man-Rogosa-Sharpe agar for mesophilic lactobacilli; M17, medium 17 agar for mesophilic lactococci; TSA, tryptic soy agar for spore-forming bacteria; PAB, Pseudomonas agar base for pseudomonads; MSA, mannitol salt agar for staphylococci; VRBGA, violet red bile salt glucose agar for Enterobacteriaceae; VRBA, violet red bile salt agar for coliforms; EHA, hektoen enteric agar for *Salmonella* spp. and *Shigella* spp. P1–P4, samples of olive fruit from four separate production batches. S1–S4, samples of brine corresponding to the same batches. *p* value: *, *p* < 0.05; **, *p* < 0.01; ***, *p* < 0.001; n.s., not significant. Data within a column followed by the same letter are not significantly different according to Tukey’s test.

**Table 2:** Phenotypic grouping of bacteria isolated from Sant’Agostino table olives.

Characters	Clusters (Number of Isolates)				
**LAB**	I (n = 36)	II (n = 8)	III (n = 12)	IV (n = 2)	V (n = 4)
Cell morphology	Coccus	Coccus (short chain)	Coccus (short chain)	Coccus (short chain)	Rod
Growth:					
▪ 15 °C	+	−	+	+	+
▪ 45 °C	+	−	−	−	−
▪ pH 9.2	+	−	−	+	+
▪ 6.5% NaCl	+	+	+	+	+
CO_2_ from glucose	−	+	+	+	−
Pentose fermentation	n.d.	n.d.	n.d.	n.d.	+
**Spore-forming bacteria**	VI (n = 3)	VII (n = 6)	VIII (n = 3)	IX (n = 6)	
Cell morphology	Rod	Rod	Rod	Rod	
Resistance to:					
▪ NaCl (%)	11	8	5	10	
▪ pH	4−10	5−10	5−9	5−9	
▪ 15 °C	+	+	+	+	
▪ 45 °C	+	−	−	+	
Glucose utilization	+	+	+	+	
Glucose fermentation	−	−	−	−	
Starch hydrolysis	+	−	+	−	

Note: LAB, Lactic acid bacteria; n.d., not determined; +, positive response; −, negative response. I–V, phenotypic groups of lactic acid bacteria. VI–IX, phenotypic groups of spore-forming bacteria.

**Table 3:** Molecular identification of bacteria strains in Sant’Agostino table olives through PCR-amplified products of 16S rDNA.

Strains	Species	% Similarity (Accession No. of Closest Relative) by		Sequence Length (bp)	Accession Number
Lactic acid bacteria		BLAST	EzTaxon		
OL40	*Enterococcus faecium*	99.86 (CP144273.1)	99.79 (AJ301830)	1428	PQ573832
OL46	*Enterococcus faecium*	99.46 (MT000137.1)	99.05 (AJ301830)	1475	PQ573833
OL53	*Leuconostoc pseudomesenteroides*	99.93 (LC223100.1)	99.51 (AB023237)	1455	PQ573834
OL55	*Lactiplantibacillus pentosus*	99.46 (OR502270.1)	99.12 (AZCU01000047)	1484	PQ573835
OL58	*Lactiplantibacillus plantarum*	99.10 (ON197164.1)	99.03 (CP032751)	1440	PQ573836
OL59	*Leuconostoc mesenteroides* subsp. *jonggajibkimchii*	99.86 (MT597785.1)	99.93 (CP014611)	1394	PQ573837
OL60	*Enterococcus faecium*	99.51 (OR016181.1)	99.16 (AJ301830)	1435	PQ573838
OL62	*Leuconostoc mesenteroides* subsp. *cremoris*	99.93 (OM265424.1)	99.50 (ACKV01000113)	1408	PQ573839
OL68	*Leuconostoc mesenteroides* subsp. *jonggajibkimchii*	99.93 (MK774577.1)	99.93 (CP014611)	1454	PQ573840
OL127	*Enterococcus faecium*	100 (CP144273.1)	99.80 (AJ301830)	1470	PQ573841
**Spore-forming bacteria**					
OL9	*Peribacillus frigoritolerans*	99.93 (MH910209.1)	99.86 (AM747813)	1401	PQ574041
OL10	*Peribacillus frigoritolerans*	99.86 (MN710443.1)	99.93 (AM747813)	1417	PQ574042
OL14	*Bacillus velezensis*	99.93 (ON197102.1)	99.79 (AY603658)	1523	PQ574043
OL16	*Peribacillus simplex*	99.87 (OP986040.1)	99.86 (BCVO01000086)	1496	PQ574044
OL17	*Priestia endophytica*	99.93 (OK605862.1)	99.93 (AF295302)	1435	PQ574045
OL19	*Priestia endophytica*	99.93 (OK605857.1)	99.93 (AF295302)	1433	PQ574046

**Table 4:** Macroscopic characters and phenotypic groups of yeasts isolated from Sant’Agostino table olives.

Macroscopic Characteristics	Clusters (Number of Isolates)		
	VI (n = 50)	VII (n = 1)	VIII (n = 3)
Colony colour ^1^	White	Tannish-white	Cream
Shape	Round	Round	Round
Edge	Fringed	Entire	Entire
Opacity	Opaque	Shiny	Opaque
Surface	Smooth	Glistening	Smooth
Consistency	Butyrous	Butyrous	Butyrous

Note: ^1^ The colony colour was determined on Wallerstein Laboratory (WL) Nutrient agar.

**Table 5:** Molecular identification of yeasts isolated from Sant’Agostino table olives.

Phenotypic Group	Amplicon Size 5.8S-ITS (bp)	Size of Restriction Fragments (bp)			Number of Strains ^1^	Species	Range Size of the PCR Products (bp)	Acc. No. (% Similarity)
		**C** ***fo*** **I**	**H** ***ae*** **III**	**H** ***inf*** **I**				
VI	550	285 + 255	460 + 85	270 + 270	4	*Candida tropicalis*	468–532	PQ574034-PQ574037 (100)
VII	805	335 + 220 + 145 + 105	805	410 + 390	1	*Torulaspora delbrueckii*	477	PQ574038 (100)
VIII	850	380 + 330 + 140	325 + 230 + 175 + 140	365 + 360 + 130	1	*Saccharomyces cerevisiae*	540	PQ574039 (100)

Note: ^1^ Determined by RAPD-PCR.

**Table 6:** Distributions of cultivable LAB, spore-forming bacteria and yeast species among Sant’Agostino table olives.

	Fruit Samples				Brine Samples			
Lactic acid bacteria	P1	P2	P3	P4	S1	S2	S3	S4
*Enterococcus faecium*		■	■	■	■	■	■	■
*Lactiplantibacillus pentosus*						■		
*Lactiplantibacillus plantarum*	■						■	
*Leuconostoc mesenteroides* subsp. *cremoris*	■		■		■	■	■	
*Leuconostoc mesenteroides* subsp. *jonggajibkimchi*		■	■	■				■
*Leuconostoc pseudomesenteroides*					■			
Spore-forming bacteria								
*Bacillus velezensis*						■		
*Peribacillus frigoritolerans*					■			■
*Peribacillus simplex*							■	
*Priestia endophytica*							■	■
Yeasts								
*Candida tropicalis*	■	■	■	■	■	■	■	■
*Saccharomyces cerevisiae*		■	■	■				
*Torulaspora delbrueckii*					■			

Note: ■ = species detected.

## Data Availability

The original contributions presented in the study are included in the article, further inquiries can be directed to the corresponding author.

## Supplementary Materials

The following supporting information can be downloaded at: https://www.mdpi.com/article/10.3390/foods14213689/s1, Figure S1: Ingredients and finished product of the traditional processing of Sant’Agostino table olives flavoured with wild fennel. (a) Wild fennel (*Foeniculum vulgare*) used as a natural flavouring; (b) Sant’Agostino cultivar table olives before processing, harvested at different stages of maturation; (c) glass jar containing olives processed according to the traditional Apulian method with the addition of wild fennel.
