# Supplementary material for: Polyphasic Characterisation of Microbiota Associated with Sant’Agostino Table Olives Flavoured with Foeniculum vulgare
Source: Foods. 2025 Oct 29;14(21):3689. doi: 10.3390/foods14213689 (PMC12610113; doi:10.3390/foods14213689)
Supplement: Supplementary file 1 [file foods-14-03689-s001.zip › foods-3949187-supplementary.pdf]

foods

MDPI

Article

Polyphasic Characterisation of Microbiota Associated with Sant'Agostino Table Olives Flavoured with *Foeniculum vulgare*

Antonio Alfonzo [1], Raimondo Gaglio [1], Davide Alongi [1], Elena Franciosi [2], Giulio Perricone [1], Giuliana Garofalo [1], Rosario Prestianni [1], Vincenzo Naselli [1], Antonino Pirrone [1], Nicola Francesca [1,*], Giancarlo Moschetti [1] and Luca Settanni [1]

1 Department of Agricultural, Food and Forest Sciences (SAAF), University of Palermo, Viale delle Scienze Bldg. 5 Ent. C, 90128 Palermo, Italy; antonio.alfonzo@unipa.it (A.A.); raimondo.gaglio@unipa.it (R.G.); davide.alongi@unipa.it (D.A.); giulio.perricone@unipa.it (G.P.); giuliana.garofalo01@unipa.it (G.G.); rosario.prestianni@unipa.it (R.P.); vincenzo.naselli@unipa.it (V.N.); antonino.pirrone@unipa.it (A.P.); giancarlo.moschetti@unipa.it (G.M.); luca.settanni@unipa.it (L.S.)

2 Research and Innovation Centre, Edmund Mach Foundation, Via Edmund Mach 1, 38010 San Michele all'Adige, Italy; elena.franciosi@fmach.it

* Correspondence: nicola.francesca@unipa.it

Figure S1. Ingredients and finished product of the traditional processing of Sant'Agostino olives flavoured with wild fennel. **(a)** Wild fennel (Foeniculum vulgare) used as a natural flavouring; **(b)** Sant'Agostino cultivar table olives before processing, harvested at different stages of maturation; **(c)** glass jar containing olives processed according to the traditional Apulian method with the addition of wild fennel.

 https://doi.org/10.3390/foods14213689

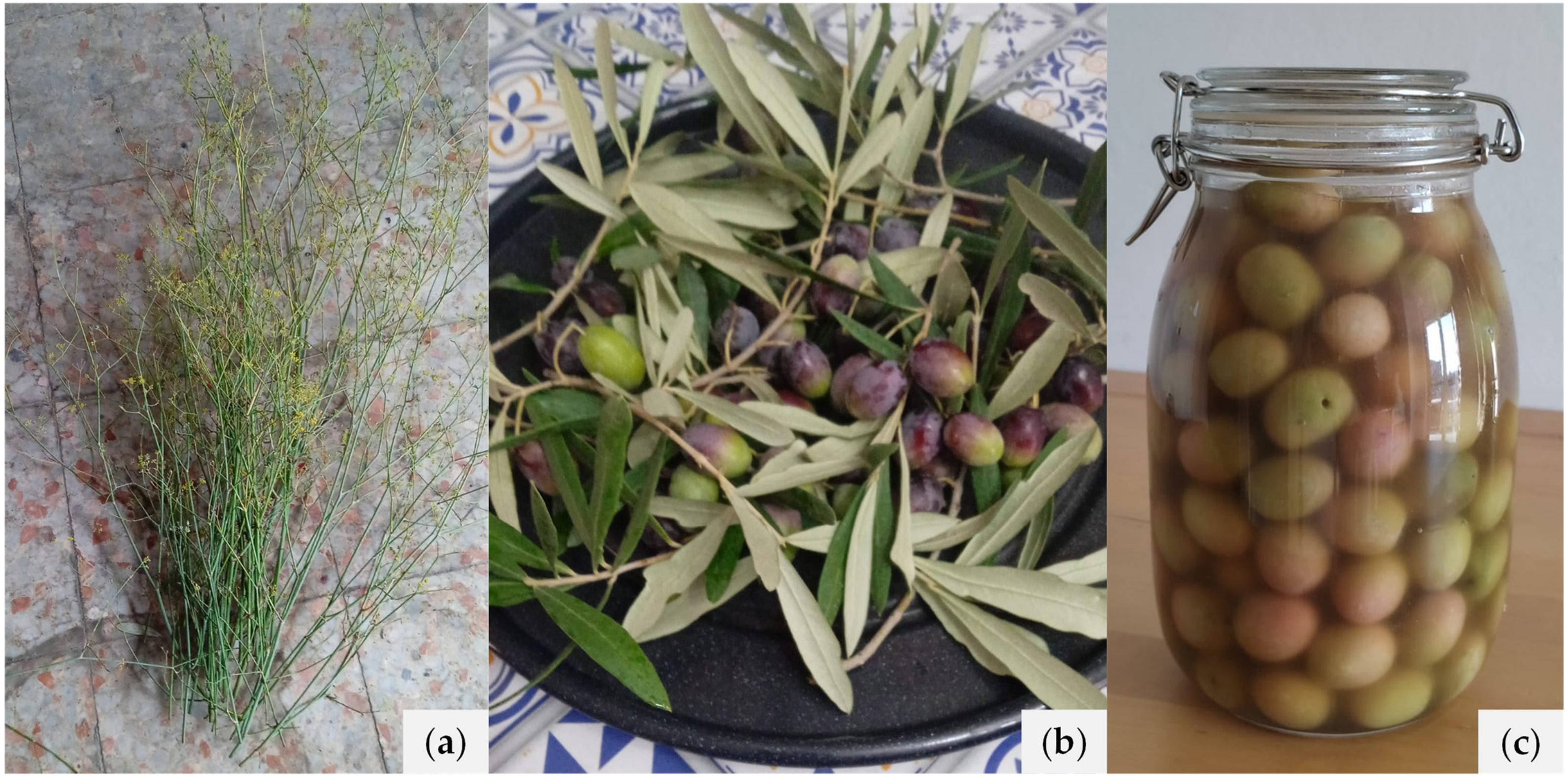

Figure S1. Ingredients and finished product of the traditional processing of Sant'Agostino table olives flavoured with wild fennel. **(a)** Wild fennel (*Foeniculum vulgare*) used as a natural flavouring; **(b)** Sant'Agostino cultivar table olives before processing, harvested at different stages of maturation; **(c)** glass jar containing olives processed according to the traditional Apulian method with the addition of wild fennel.
